# Modulation of Visual Cortex Excitability by Continuous Theta Burst Stimulation Depends on Coil Type

**DOI:** 10.1371/journal.pone.0159743

**Published:** 2016-07-26

**Authors:** Sabrina Brückner, Thomas Kammer

**Affiliations:** Section for Neurostimulation, Department of Psychiatry, University of Ulm, Ulm, Germany; University Medical Center Goettingen, GERMANY

## Abstract

Subthreshold continuous theta burst stimulation of the visual cortex has been reported to cause inhibitory effects on phosphene threshold. In contrast, we observed no inhibition in a former study applying higher stimulation intensities. The main discrepancies between our experiments and the former studies were stimulation intensity and coil type. We aimed at investigating the role of these factors on the modulatory effects of continuous theta burst stimulation applied to the visual cortex. In a between-group-design, we used either a figure-of-eight-coil or a round coil, respectively. We measured phosphene thresholds prior and after continuous theta burst stimulation applied at 80% of individual phosphene threshold. With the figure-of-eight-coil, phosphene thresholds significantly decreased following stimulation. This is in line with the results of our former study but contrary to the increase observed in the other two studies. Using a round coil, no significant effect was observed. A correlation analysis revealed an inhibitory effect in subjects with higher phosphene thresholds only. Furthermore, the slope of the baseline phosphene threshold seems to predict the direction of modulation, independent from coil type. Thus, modulatory effects of continuous theta burst stimulation seem to depend on coil type and psychophysics parameters, probably due to different cortex volumes stimulated. Stochastic resonance phenomena might account for the differences observed.

## Introduction

Phosphenes, defined as elementary visual percepts, can be evoked by pulses of transcranial magnetic stimulation (TMS) applied to the occipital pole (e.g., [1–3]). The minimum TMS intensity required to elicit phosphenes is called phosphene threshold (PT). Indicating the individual visual cortex excitability of the stimulated area, PT is very stable across multiple sessions showing high test-retest-reliability (e.g., [4–9]). In several studies, PT was used as the dependent variable to investigate changes in visual cortex excitability. For instance, it was shown that the pattern of light deprivation has a substantial impact on visual cortex excitability. Whereas binocular light deprivation causes an increase in visual cortex excitability as indicated by decreased phosphene thresholds [4], the opposite was true for monocular light deprivation [10]. Furthermore, PT measurements provide evidence for the inferred hyperexcitability in the occipital cortex in migraine showing lower phosphene thresholds in migraineurs compared to healthy subjects [11]. Lower PTs demonstrated increased visual cortex excitability following a period of reading [12]. In contrast, PTs increased with larger background contrasts [13], indicating modulation of visual cortex excitability in dependence of a specific visual input.

Moreover, changes in phosphene threshold are used to investigate the effects of neuromodulatory techniques such as repetitive TMS (rTMS) protocols on visual cortex excitability. For instance, the inhibitory effect of 1Hz rTMS known from the motor cortex revealed by decreased motor evoked potentials as well as increased motor thresholds [14–17] was successfully transferred to the visual system. Here, several studies showed increased PTs following 1Hz rTMS [18–20]. Likewise, the aftereffects of transcranial direct current stimulation (tDCS) showed the same pattern for both the motor cortex [21, 22] and the visual cortex [7], namely excitation with anodal tDCS and inhibition with cathodal tDCS. Importantly, whereas in the visual cortex phosphene thresholds were modulated by tDCS [7], in the motor system other parameters like the input-output-curve changed [23]. However, motor thresholds remained stable [21–23]. These findings suggest that neuromodulation techniques may act in a different way in the two cortical areas. Therefore, results of the motor system are not directly transferable to the visual system. This is strengthened by the fact that motor thresholds and occipital PTs [5, 6] as well as motor thresholds and moving PTs evoked over V5/MT [24] were shown to be uncorrelated. It was suggested that this could be due to anatomical differences between neuronal elements in the cortical areas, or differences in size and orientation of the neurons and the cortical network properties controlled by different neuromodulators [24]. Nevertheless, when continuous theta burst stimulation (cTBS) was shown to have an inhibitory effect on the motor cortex [25], this modulation was successfully transferred to the visual cortex [26]. In contrast, in a recent study we were not able to directly modulate PTs applying TBS with an intensity of 100% of individual phosphene threshold [9]. Surprisingly, we found a numerical decrease in PTs following cTBS, although not reaching statistical significance. However, another study replicated the inhibitory effect on PTs using cTBS [27]. Thus, there are at least two independent studies showing increased phosphene thresholds following cTBS applied either at 80% of individual PT [26] or at 80% of active motor threshold [27]. Since subthreshold stimulation can reverse TMS effects [28], the observed trend to decreased PTs following cTBS [9] could be due to the higher intensity (100% PT). Another main difference between our study [9] and former publications [26, 27] is the coil type used in those studies. They showed an inhibitory effect on PTs by using a round coil, whereas the tendency to decreased PTs in our study was observed using a figure-of-eight-coil. Since figure-of-eight-coils are more focal than circular coils [29], TBS to the motor cortex is commonly applied using a figure-of-eight-coil [25, 30–32]. For the visual system, both round coils [26, 27] as well as figure-of-eight-coils [9, 33–35] are used for TBS application.

Recently, it was suggested that stochastic resonance effects reveal the neural mechanisms of TMS [36]. Stochastic resonance is described as the enhancement of the response of a system to a weak signal by noise [37]. Supposing that TMS acts by adding noise to neuronal processing [36], stochastic resonance phenomena may also explain the discrepancies between our experiments [9] and former studies [26, 27].

In the present study, we explored the aftereffects of cTBS applied to the visual cortex on phosphene thresholds using either a round coil or a figure-of-eight-coil. To exclude TBS intensity as a source for the different effects described above, we used 80% of the individual PT as in the original study [26] to replicate the results. We hypothesized that, in accordance with the former studies [26, 27], application of cTBS with a round coil would increase phosphene thresholds. For the figure-of-eight-coil group, due our previous findings contradicting the canonical modulation, we had no clear hypothesis. An increase in PT observed in the present experiment would indicate that the trend to decreased PTs in our former study [9] was caused by the application of higher stimulation intensities. If PTs would be decreased again following cTBS with the figure-of-eight-coil, the reason for the different effects observed should be the coil type.

## Material and Methods

### Subjects

Thirty subjects were recruited for the study. All of them had participated in at least one former study in our lab and were selected due to their stability in phosphene thresholds. Exclusion criteria were metallic implants, prior history of neurological or psychiatric disorders, chronic tinnitus, major medical illness, drug abuse or alcoholism, any medication with the exception of contraceptives. The subjects were divided into two groups (15/15) being stimulated with either a figure-of-eight-coil (8 male, mean age 24.5±3.6 years) or a round coil (8 male, mean age 23.2±2.0 years). All participants gave their written informed consent and were paid for participation. The study followed the Declaration of Helsinki and was approved by the Ethics Committee of the University of Ulm.

### Experimental design

During the whole session subjects sat in a comfortable chair in normal ambient light. The chair was located at the same position for every subject. During the whole experiment (for PT determinations as well as for TBS application) they kept their eyes open looking at a white wall. This was done to keep visual input the same for all subjects since different inputs would cause different neural activation states in the visual cortex [38]. Subjects were stimulated with biphasic magnetic pulses delivered with a Magpro X100 stimulator (MagVenture, Farum, Denmark) and either a figure-of-eight-coil (MC-B70) or a round coil (MC125). In the figure-of-eight-coil group, the coil position in relation to the head was monitored, registered and maintained with the frameless stereotactic positioning system BrainView (V2, Fraunhofer IPA, Stuttgart, Germany, cf. [39]). In the round coil group, the coil position was marked on the scalp and kept constant manually by the experimenter on the hot-spot position.

**Fig 1:**
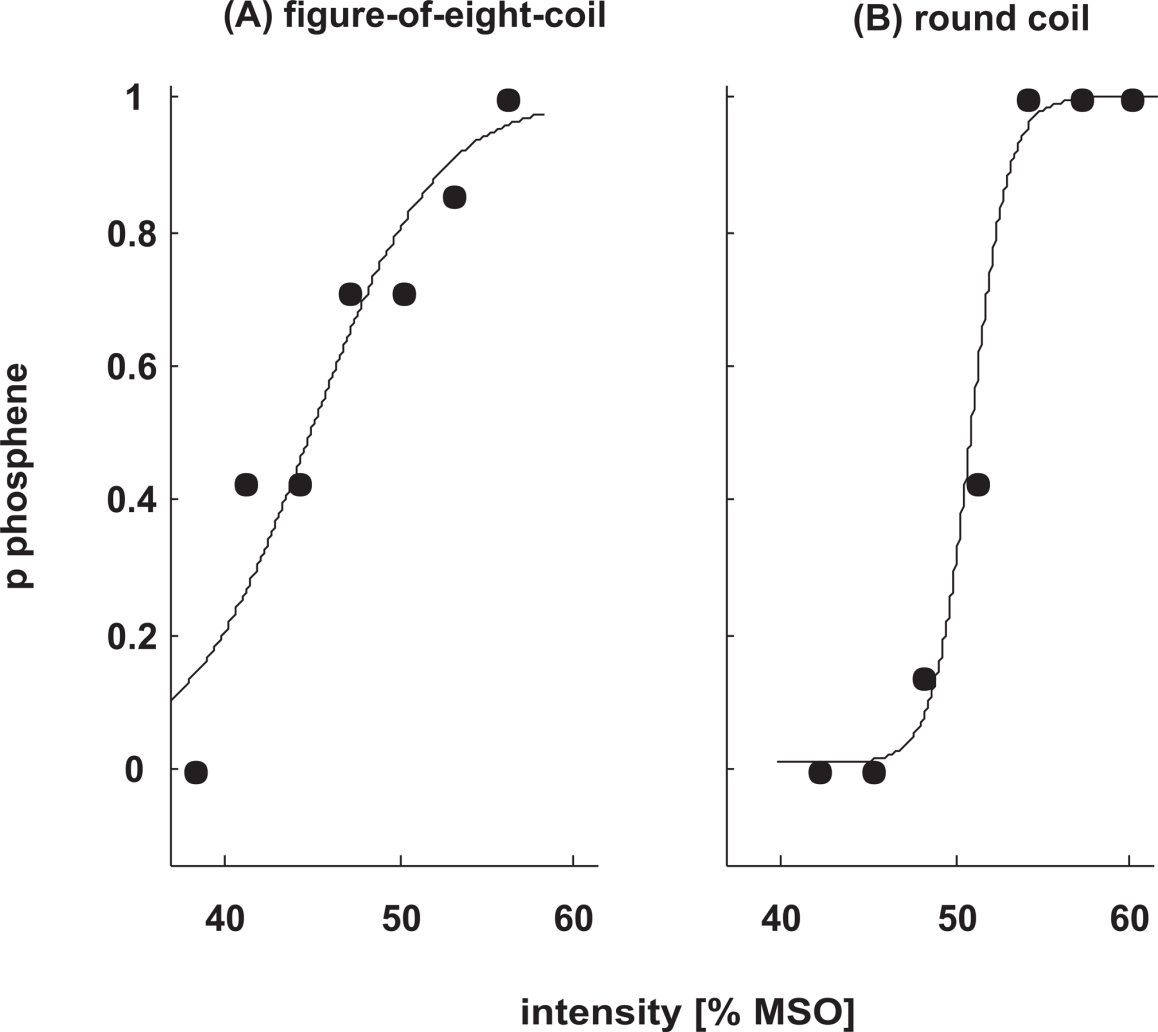
Examples of sigmoidal fits applied to the responses in a PT measurement. **(A):** one subject of the figure-of-eight-coil group (threshold 44.9, slope 3.6) **(B):** one subject of the round coil group (threshold 50.9, slope 1.1).

All subjects had participated in at least one experiment in our lab before, including a familiarization procedure where they were trained to observe phosphenes. Therefore, phosphene hot-spots as well as approximate phosphene thresholds of the subjects were available. At the beginning of the session, using the figure-of-eight coil we re-established a phosphene hot spot based on the coil position registered in a former study. Stimulation intensity was 110% of former phosphene threshold. Whereas in most of the subjects a strong phosphene was perceived with the former hot spot, in some subject a small change of coil position as well as a small rotation of the coil yielded a clearer percept of phosphenes compared to the formerly registered coil position. In these cases we used the corrected hot spot for the entire experiment. In the round coil group, the coil was positioned with the lower rim over the hot spot registered in a former study. The handle was oriented upwards, and the B-face pointing to the experimenter (cf. [26]). For both coil types, in most cases the final coil position was located on the left hemisphere, with the second phase of the magnetic pulse inducing a current in the brain with a medio-lateral direction. Phosphene thresholds were then measured following a previously established protocol (cf. [40]). The procedure was identical to that published previously [9]. In brief, method of constant stimuli was applied at 7 levels with 7 repetitions each in a randomized manner, with at least 5 seconds between pulses. The approximate duration of one PT determination was 5 minutes. Like in our former study, a sigmoidal fit applied to the responses estimated the individual PT at the reversal point (alpha) of the logistic function, as well as the slope (beta) of the function (psignifit, [41]). The lower and upper borders, gamma and 1 –lambda, were fixed to 0 and 1. A smaller beta value indicates a steeper slope. An example is shown in Fig 1 for each group, respectively.

In the figure-of-eight-coil group, PT was initially measured once. In the round coil group, PT was measured twice, since the first PT measurement was taken as practice run and discarded. This practice run was performed because none of the subjects was ever stimulated with a round coil before. Subsequently, cTBS was applied to the predetermined phosphene hot-spot at 80% of individual PT. For cTBS, the same coil was used as for the PT measurements. Two minutes (post 1) and 12 minutes (post 2) after the end of TBS, PT was estimated again using the same range of intensities as in the baseline measurements.

In a replication experiment, 12 further subjects (6 male, mean age 23.4±2.0 years) who all had participated in at least one former study in our lab, but not in the main experiment of this study, were selected due to their stability in phosphene thresholds. In this separate group, cTBS was delivered over the visual cortex, using the figure-of-eight-coil. The experimental design was the same as in the main experiment, but the post 2 PT measurement was omitted.

### Data analysis

PT values were analyzed regarding normal distribution beyond all subjects of the main experiment for the three time points separately. Pre and post cTBS phosphene thresholds were subjected to repeated-measures analyses of variance (rmANOVAs; Statistica V.12, StatSoft GmbH, Hamburg, Germany). Sphericity requirements were assessed using Mauchley’s test. There was no violation, thus correction was dispensable. Post-hoc analyses were performed using Newman-Keuls test. Baseline PTs and slopes of the sigmoidal fits generating the thresholds were compared between the groups using Student’s t-test. A correlation analysis for baseline PTs and size of cTBS effects was performed by calculating Pearson’s correlation coefficients for the two groups separately.

## Results

### Main analysis

Shapiro- Wilk’s test revealed that the data follows normal distribution (pre: W = 0.98, p = 0.84; post 1: W = 0.99, p = 0.96; post2: W = 0.98, p = 0.72). Mean baseline PT value was 41.1±9.9% maximum stimulator output (MSO) in the figure-of-eight-coil group and 44.6±7.7% MSO in the round coil group, respectively. Baseline PTs were numerically higher in the round coil group compared to the figure-of-eight-coil group, but the difference did not reach significance as revealed by Student’s t-test (t = -1.06, p = 0.297).

**Fig 2:**
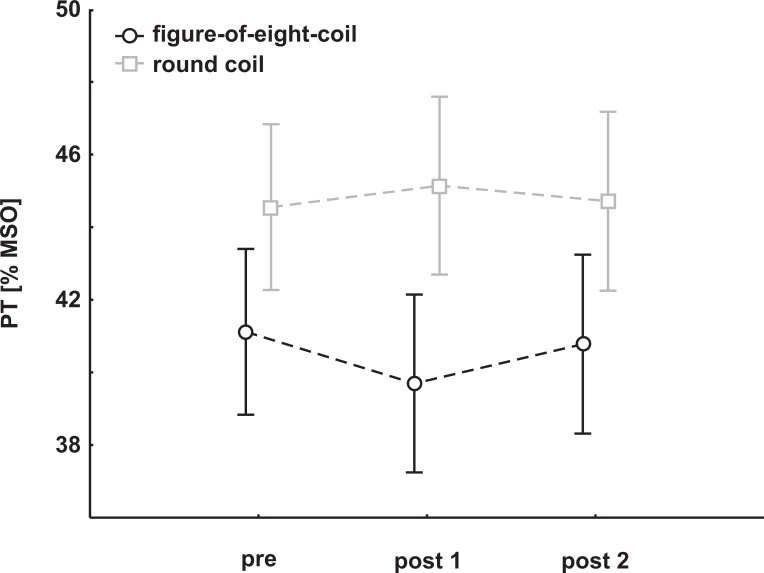
cTBS effects on PTs. Mean group PT change (±SEM) following cTBS. An rmANOVA with the within-factor PREPOST (pre/post 1/post 2) and the between-factor COIL (figure-of-eight-coil, round coil) revealed no main effect and no interaction. Omitting the post 2 measurement, PTs decreased significantly in the figure-of-eight-coil group.

The pre and post PT values of all subjects were subjected to an rmANOVA with the within-factor PREPOST (pre, post 1, post 2) and the between-factor COIL (figure-of-eight-coil, round coil). Neither main effects (PREPOST: F_(2,56)_ = 0.43, p = 0.65; COIL: F_(1,28)_ = 1.63, p = 0.21) nor interactions (PREPOST x COIL: F_(2,56)_ = 2.46, p = 0.095) were found. Mean group data are depicted in Fig 2.

Due to the trend to a significant interaction, we decided to discard the post 2 measurement for further analysis, as our cTBS effect tended to be present only a few minutes.

Pre and post 1 PT values were subjected to an rmANOVA with the within-factor PREPOST (pre, post 1) and the between-factor COIL (figure-of-eight-coil, round coil). No main effect was found (PREPOST: F_(1,28)_ = 0.89, p = 0.35; COIL: F_(1,28)_ = 1.79, p = 0.19), but a significant interaction (PREPOST x COIL: F_(1,28)_ = 5.24, p = 0.03). Post hoc Newman Keuls test revealed a significant decrease of PTs in the figure-of-eight-coil group (pre mean: 41.1% MSO, post 1 mean 39.7% MSO; p = 0.03), whereas no significant differences in the round coil group were observed (pre mean: 44.6% MSO, post 1: 45.1% MSO; p = 0.35).

Since we observed small effects of cTBS on PT with a figure-of-eight-coil only, pointing in the “wrong” direction, we assumed to identify subgroups in the response to cTBS when analyzing the data individually.

### Explorative analysis

**Fig 3:**
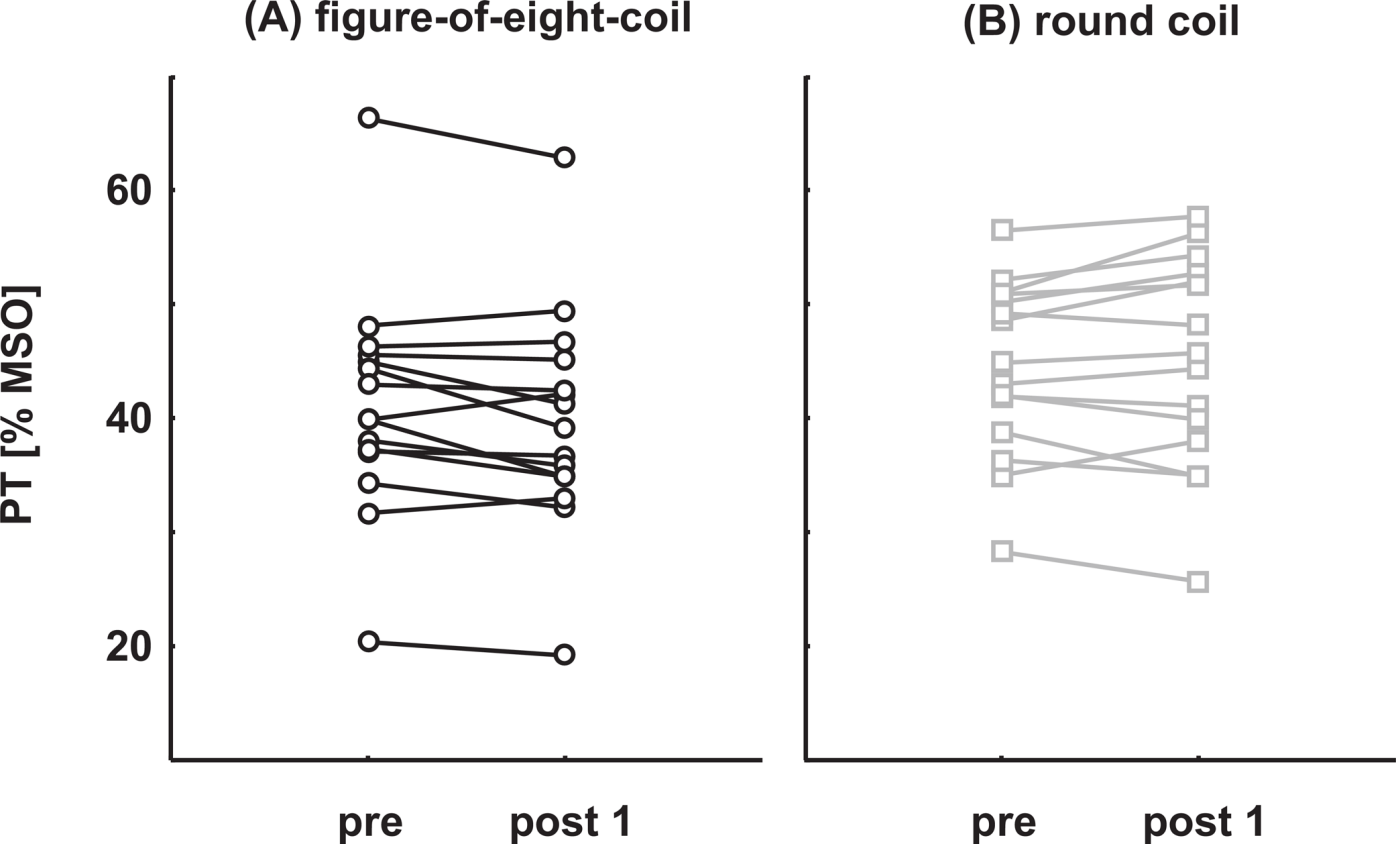
Individual effects of cTBS on PTs. **(A):** figure-of-eight-coil group **(B):** round coil group.

Individual pre and post 1 PT values are shown in Fig 3 for the two groups separately. In the figure-of-eight-coil group, 11 out of 15 subjects showed a (numerical) decrease in PT following stimulation. In the round coil group, in 9 out of 15 subjects PT increased after cTBS. The remaining subjects showed the reversed pattern.

**Fig 4:**
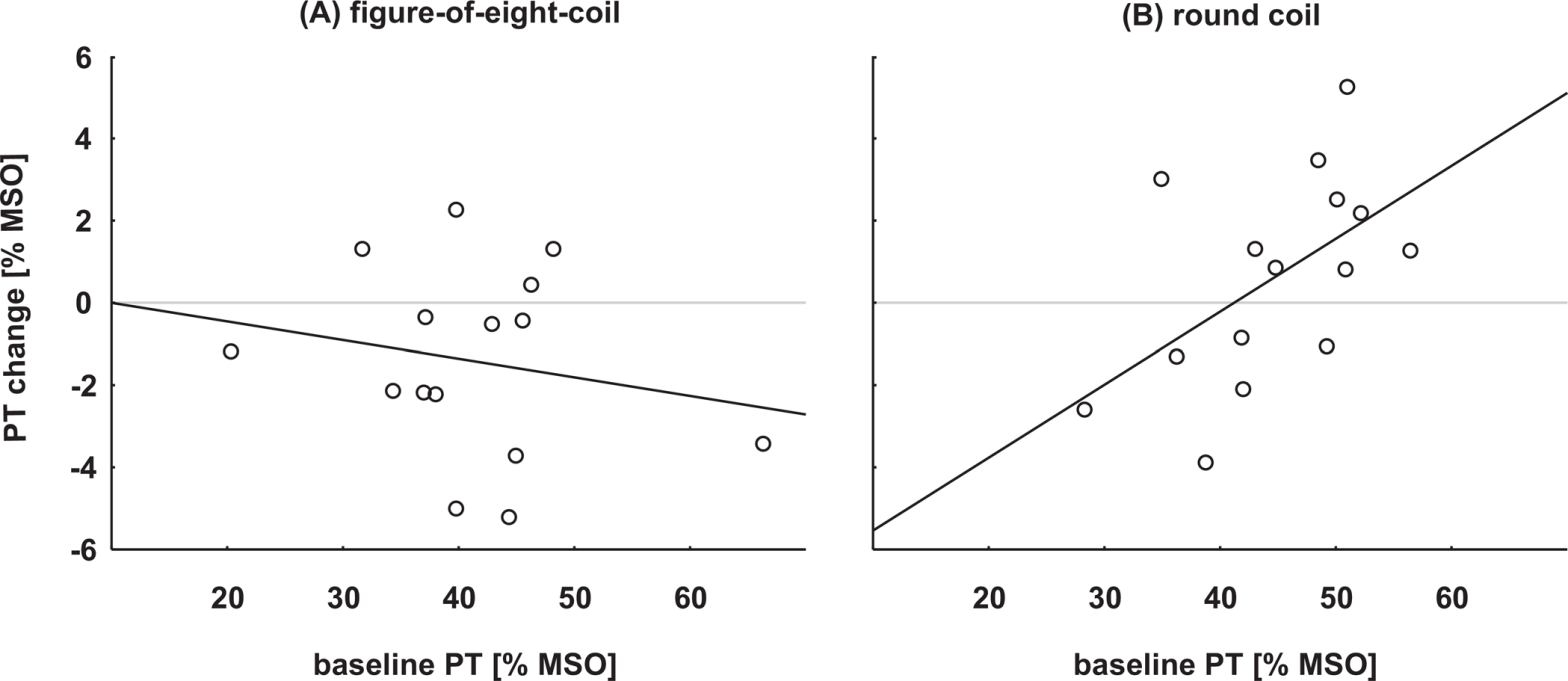
Correlation of baseline PTs and cTBS effects. **(A):** figure-of-eight-coil group (n = 15, no correlation, r = -0.19, p = 0.49) **(B):** round coil group (n = 15, significant positive correlation, r = 0.54, p = 0.04).

Since the data suggested that cTBS effects might depend on baseline PTs, we correlated baseline PT and size of cTBS effect (post 1 –pre value). For the figure-of-eight-coil group, no correlation was observed (r = -0.19, p = 0.49). For the round coil group, we found a significant positive correlation (r = 0.54, p = 0.04) indicating that subjects with higher baseline PTs showed larger inhibitory cTBS effects. As depicted in Fig 4, subjects with lower baseline PTs tended to excitatory effects following cTBS applied with a round coil.

### Slopes

Phosphene threshold values were estimated applying a sigmoidal fit (psignifit, [41]) to the subject’s responses, with the reversal point of the logistic function as individual PT. The slopes of the estimated functions were steeper in the round coil group compared to those of the figure-of-eight-coil group as indicated by a Student’s t-test (t = 2.30, p = 0.03).

### Replication experiment

The observation in our main experiment, namely the excitatory effect of cTBS under certain conditions, is in contrast to findings observed by at least two independent studies previously published [26, 27]. The aim of the replication experiment was to confirm our finding of significant decreased phosphene thresholds following cTBS applied at 80% of the individual PT using a figure-of-eight-coil.

Mean baseline PT value was 44.1±10.4% MSO. Data were normally distributed as revealed by Shapiro- Wilk’s test (pre: W = 0.96, p = 0.76; post 1: W = 0.96, p = 0.78). Baseline PTs of this group were not different from those of the figure-of-eight-coil group in the main experiment (Student’s t-test, t = 0.77, p = 0.45). A paired t-test applied to the pre and post PT values in the replication experiment revealed no effect of cTBS on phosphene thresholds (t = 0.51, p = 0.62).

**Fig 5:**
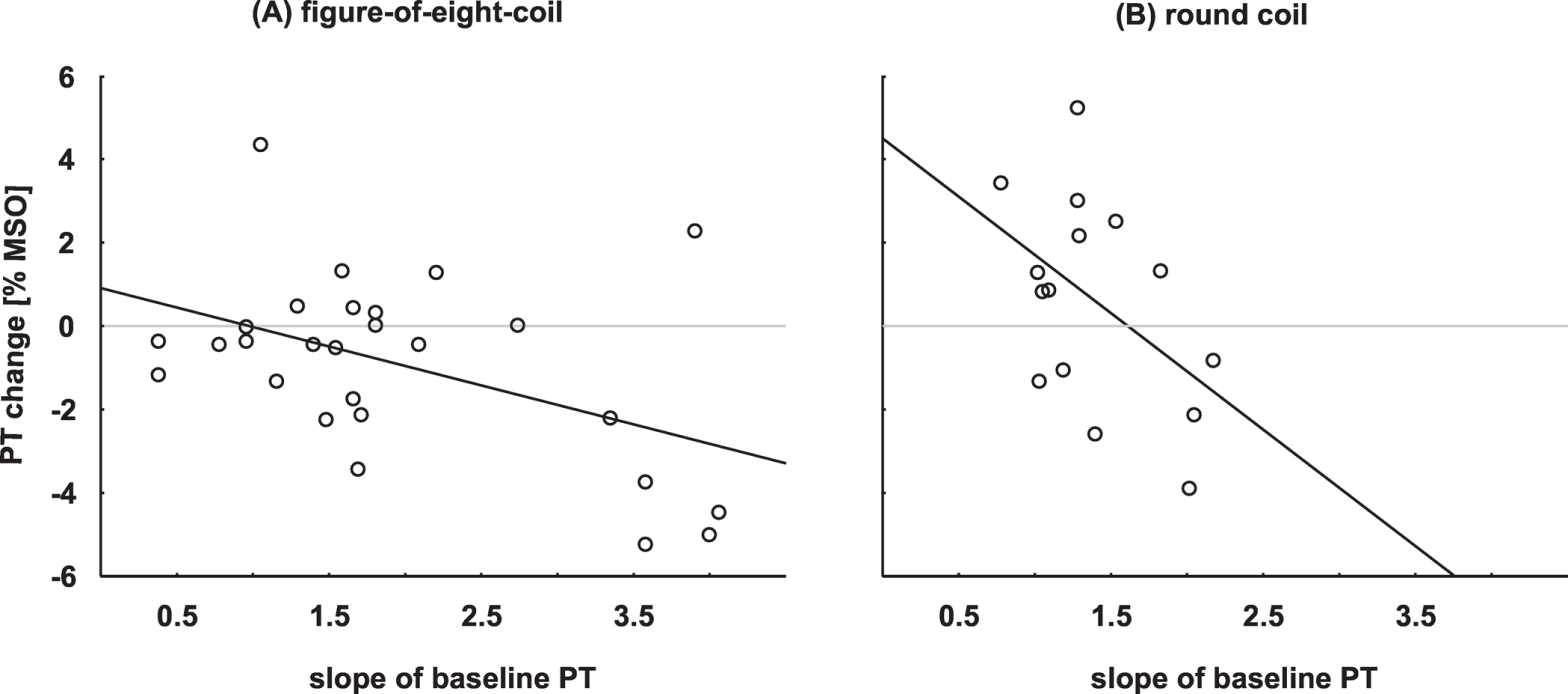
Correlation of the slope of baseline PTs and cTBS effects. **(A):** figure-of-eight-coil group (n = 27, significant negative correlation, r = -0.47, p = 0.01) **(B):** round coil group (n = 15, trend to significant negative correlation, r = -0.47, p = 0.07). Note that smaller values indicate steeper slopes.

Finally, we pooled the 12 subjects of the replication experiment and the 15 subjects of the figure-of-eight-coil group of the main experiment. The data followed normal distribution (pre: W = 0.98, p = 0.85; post 1: W = 0.98, p = 0.83). A paired t-test revealed that the significant decrease of phosphene thresholds following cTBS observed in the main experiment was still significant for the larger sample size (t = 2.18, p = 0.039). Pearson’s correlation analysis again revealed no correlation between baseline PT and size of cTBS effect (r = -0.15, p = 0.46) in the figure-of-eight-coil group. However, there was a significant correlation between the slope of the baseline PT and size of cTBS-effect (r = -0.47, p = 0.01), indicating that subjects with flatter slopes in their PT function showed larger facilitatory cTBS effects. For the round coil group, Pearson’s correlation analysis revealed a trend between baseline PT slope and size of cTBS effect (r = -0.47, p = 0.07) in the same direction (see Fig 5). Interestingly, baseline PT and slope of baseline PT are not correlating with each other (figure-of-eight-coil group: r = 0.04, p = 0.85; round coil group: r = -0.32, p = 0.24) and no significant difference was found comparing the slopes of pre and post PTs (figure-of-eight-coil group: t = 1.28, p = 0.21; round coil group: t = 0.15, p = 0.88).

## Discussion

We investigated the effects of cTBS applied with 80% of the individual phosphene threshold on visual cortex excitability. In a between-group-design, we used either a round coil or a figure-of-eight-coil for TBS as well as for PT determination. The observed change of PT was highly variable across the subjects, but we found a short-lasting significant increase in visual cortex excitability in the figure-of-eight-coil-group. In a replication experiment, this excitatory effect could not be observed. When we pooled the subjects of the two figure-of-eight-coil experiments, the excitatory effect remained significant. The high interindividual variability observed in cTBS effects correlated with the slope of the baseline PT, thus flatter slopes tend to predict facilitatory cTBS effects. Furthermore, for the round coil group cTBS effects were positive correlated with baseline PTs. Thus, our results are in contrast to two former studies [26, 27] showing decreased visual cortex excitability following cTBS applied with a round coil.

In our former study we investigated the effects of TBS on phosphene thresholds in dependency of visual demand using a visual acuity task following stimulation [9]. We observed a significant increase in PT only in the condition with high visual demand following cTBS, but no change in PT in the condition with low visual demand. The direct effect of cTBS alone was just a numerical decrease of phosphene thresholds. Thus, our observation was in contrast to the inhibitory effect reported before [26]. Since another research group was able to replicate their results [27], we identified two main differences potentially being the source for the discrepancies between the results. First, in our experiment [9] we applied cTBS with 100% of the individual PT whereas the other two studies used subthreshold intensities with either 80% of the PT [26] or 80% of the active motor threshold [27]. Second, we applied TBS with a figure-of-eight-coil instead of a round coil. Thus, in the current experiment we used 80% of the individual PT as TBS intensity and both coil types. As we observed decreased PTs following cTBS in the figure-of-eight-coil group like in our former study, the intensity is unlikely to be responsible for the conflicting results. Merely, the subthreshold 80% cTBS led to an excitatory effect on PTs even more pronounced than the 100% cTBS intensity in our former study. Instead, coil type seems to influence the effects of cTBS on visual cortex excitability. Using a round coil, we found highly variable effects on PTs but a positive correlation of baseline PT and size of cTBS effect. Whereas subjects with higher baseline PTs showed the expected increase in PTs, those subjects with lower baseline PTs showed a tendency to decreased PTs following cTBS. For both coil types, we found a negative correlation between the slope of the baseline PT and size of cTBS effect, although not statistically significant in the round coil group. Since the thresholds observed in the studies are not comparable due to different magnetic stimulators, coils, experimenters and PT determination procedures, we can only speculate that the subjects of Franca et al. [26] and Allen et al. [27] showed higher PT baseline values and/or steeper slopes.

In our view the different effects of cTBS on PTs are explained best by stochastic resonance effects [36]. Stochastic resonance is commonly understood as the enhancement of the response of a system to a weak signal by noise [37]. Whereas for single threshold systems stochastic resonance effects can be observed only for subthreshold signals, multithreshold networks can display stochastic resonance effects even if the signal is suprathreshold [37]. Due to the stochastic resonance phenomenon, in a nonlinear system the injection of low level noise can lower the threshold of the system, whereas higher noise levels disrupt performance. Thus, TMS may act by adding different levels of noise to the system, explaining variability in the effects in some respects—at least in online TMS protocols [36, 42]. Assuming that the application of cTBS to the visual cortex with 80% of individual PT using a figure-of-eight-coil may add low levels of noise to the visual system, lower thresholds might be a result of stochastic resonance for most of the subjects. In our former study [9] we used 100% intensity with the very same coil and observed only a trend to decreased thresholds. This could be explained by exceeding the amount of noise that would improve signal detection in stochastic resonance framework. In addition, the visual acuity task following cTBS might have raised noise levels even more and thus led to increased phosphene thresholds [9]. The question would now be how to explain the different effect achieved with the round coil by means of stochastic resonance phenomena. Since round coils are less focal than figure-of-eight-coils [29], for a given intensity a larger volume is reached by the stimulation pulse depolarizing a higher amount of neurons. This could explain the steeper slopes of the sigmoid functions estimating phosphene thresholds. For the motor system, it was suggested that the stimulus-response-curve may be steeper for the round coil than for the figure-of-eight-coil [43] since the total descending activity for a given intensity above the threshold will be higher with the non-focal round coil [44]. However, there is a difference between applying single TMS pulses when measuring the threshold and the repetitive stimulation applying the TBS pattern. It is plausible that at PT the same amount of neurons are depolarized comparing the two coil types. But reducing the stimulation intensity to 80% of PT will reduce the volume of depolarized neurons differently. As indicated by the steeper slopes the volume under the round coil is more decreased than the volume under the figure-of-eight coil. Thus, network modulation with TBS should be different.

Individual baseline PT contributes to differences in modulatory effects, too. For subjects with a low PT, the round coil may act like the figure-of-eight-coil adding low levels of noise to the system, thus increasing excitability and lowering the threshold numerically. If a subject has a high PT, visual cortex is less excitable due to a number of reasons. For instance, there might be a larger background-activity of the neurons. This elevated baseline noise would be increased further by TBS and thus, due to the stochastic resonance phenomenon, would lead to increased PTs. But if so, one might still wonder why this is not the case for subjects with higher baseline PTs stimulated with the figure-of-eight-coil. In the motor system it was suggested that, with the round coil, neurons oriented at different angles may be stimulated and the evoked response is spread across muscles [43]. For the figure-of-eight-coil, the spread of current to other regions is more restricted [44]. For the visual system, it is also conceivable that TBS with a round coil reaches a bigger cortical area compared to TBS with a figure-of-eight-coil due to larger spreading. Thus, more neurons are involved when stimulating with a round coil repetitively, and some of these “extra- neurons” may be more excitable than those originally stimulated in the threshold- measurements. If stimulation intensity is high enough to depolarize these more distant neurons as it could be the case in subjects with high PTs, then the noise levels added by TBS could be high enough to impair the system as revealed by increased phosphene thresholds.

However, there are some studies applying TBS to the visual cortex focusing on parameters other than PT changes. For instance, Waterston & Pack [35] found an improved discrimination of certain visual features following cTBS, depending on the type of the task. They applied TBS with a focal figure-of-eight-coil and a fixed intensity of 43% MSO, observing a mean subject PT of 64.6% MSO. Thus, TBS intensity was very low and it might be possible that low noise levels were added to the visual system, leading to an improvement in dependency of the additional noise caused by the task. When Rahnev et al. [34] applied cTBS to the visual cortex with 80% of individual PT using a figure-of-eight-coil, they found decreased accuracy in a perceptual discrimination task subsequently. In contrast, in our former study we found no modulation of peripheral visual acuity following the same type of stimulation [33]. It is conceivable that the task of Rahnev et al. [34] added more noise to the system, thus decreasing performance. As we found no modulation of peripheral visual acuity applying TBS with various intensities [33], our task might have required more complex visual functions that could not be altered by TBS to the visual cortex. Finally, Allen et al. [27] observed increased PTs and, as a conflicting result, enhanced conscious vision following cTBS to the visual cortex. They concluded that stochastic resonance cannot account for their results and suggested that TBS acts most probably through gating-by-inhibition. However, they used a round coil for cTBS. The effect of cTBS on phosphene threshold and on conscious vision was investigated in two separate experiments, with only three subjects participating in both of them. Therefore, it might be conceivable that the subjects in the two experiments belonged to different subgroups regarding baseline excitability, with different directions of modulation following cTBS applied with a round coil. Since Allen et al. [27] used the motor threshold for calibration of the cTBS intensity, it is not clear whether their subjects had high or low baseline PTs.

As another source for the individual differences in response to cTBS, we observed a correlation between the slopes of the PT functions and cTBS effect, independent of the coil type used. Whereas subjects with steep slopes showed a tendency to inhibition following cTBS, subjects with flatter slopes tended to facilitation. For the figure-of-eight-coil group, flatter slopes were found compared to the round coil group, leading to a significant facilitatory cTBS effect at the group level. A flatter slope in PT function could, for instance, indicate a subject´s uncertainty in phosphene detection, higher background activity or heterogeneity in excitability of the stimulated neurons. If uncertainty is the reason, this would mean that the lower PTs in the post measurements indicate a training effect in phosphene detection rather than a cTBS effect per se. However, as a consequence of training, the slopes of the post PTs should be steeper, which was not the case. If the reason is higher background activity, it is conceivable that cTBS acts via gating-by-inhibition. Inhibition of background activity via cTBS would increase the signal-to-noise ratio, resulting in decreased PTs. However, in this case again slopes of the post PTs should be steeper. Heterogeneity in excitability of the neurons could explain why the slope of the function is not modified by cTBS although PT values changed. But why would higher heterogeneity in the neuron´s excitability would led to a facilitatory cTBS effect? It is conceivable that, in terms of stochastic resonance phenomenon, only a small part of the heterogeneous neurons are affected by cTBS resulting in a small increase of noise leading to facilitation. If subjects show more homogeneity in neuron’s excitability, then cTBS would add more noise to the system, leading to an inhibitory cTBS effect. Although the slope of the PT function seems to predict the direction of the cTBS effect, this is not the exclusive explanation of interindividual variability. Since there was no correlation between the slope and the value of baseline PTs, at least for the round coil a combination of both factors seems to predict the cTBS effect.

Of course, all these explanations are highly speculative. Nevertheless, in our view the stochastic resonance phenomenon can account for the heterogeneous results observed following cTBS applied to the visual cortex. More research in this field, especially regarding the role of different coil types and intensities is necessary to understand the modulatory mechanisms of repetitive TMS patterns.

There are two main limitations concerning our study. First, a within-group design would have improved our data. However, as many of our phosphene-experienced subjects were not available for a second session, we have chosen a between-group design. Another limitation is the lack of a control condition, for instance iTBS or sham stimulation. Since neither Franca et al. [26] nor our group [9, 33] were able to show any modulatory effect applying iTBS to the visual cortex, a comparison of the effects of iTBS applied with different coil types seems to be unnecessary. On the other hand, null findings in this experiments do not mean that there is no effect of iTBS on visual cortex at all, making it difficult to use it as a control condition. For rTMS studies, sham conditions are commonly realized rotating the coil perpendicular to the surface of the head, or using very low application intensities. Since the subject will feel the difference this way, in our view such sham conditions are not appropriate. The best method for rTMS sham conditions is the use of a sham coil providing electrical stimulation of the skin [45], thus inducing about the same sensations for the subjects as stimulation with a real coil. However, unfortunately there is no such sham coil available in our lab. In the future we are planning to compare TBS effects in the motor system using round coil and figure-of-eight-coil in a within-subject design.

## Supporting Information

S1 File Raw_data. Individual phosphene thresholds and slopes.
(XLSX)
